# Medical and Infectious Complications Associated with Pyelonephritis among Pregnant Women at Delivery

**DOI:** 10.1155/2013/124102

**Published:** 2013-09-28

**Authors:** Sarah K. Dotters-Katz, R. Phillips Heine, Chad A. Grotegut

**Affiliations:** Division of Maternal-Fetal Medicine, Department of Obstetrics and Gynecology, Duke University, DUMC Box 3967, Durham, NC 27710, USA

## Abstract

*Objective*. Pyelonephritis is a common cause of antepartum admission and maternal morbidity. Medical complications associated with pyelonephritis during delivery are not well described; thus the objective of this study was to estimate medical, infectious, and obstetric complications associated with pyelonephritis during the delivery admission. *Study Design*. We conducted a retrospective cohort study using the Nationwide Inpatient Sample (NIS) for the years 2008–2010. The NIS was queried for all delivery-related discharges. During the delivery admission, the ICD-9-CM codes for pyelonephritis were used to identify cases and were compared to women without pyelonephritis. A multivariable logistic regression model was constructed for various medical, infectious, and obstetric complications among women with pyelonephritis compared to women without, while controlling for preexisting medical conditions and demographics. *Results*. During the years 2008–2010, there were 26,397 records with a diagnosis of pyelonephritis during the delivery admission, for a rate of 2.1 per 1000 deliveries. Women with pyelonephritis had increased associated risks for transfusion, need for mechanical ventilation, acute heart failure, pneumonia, pulmonary edema, acute respiratory distress syndrome, sepsis, acute renal failure, preterm labor, and chorioamnionitis, while controlling for preexisting medical conditions. *Conclusions*. Pyelonephritis at delivery admissions is associated with significant medical and infectious morbidity.

## 1. Introduction

Pyelonephritis during pregnancy has the potential to cause serious morbidity to the pregnant woman. It is the most common nonobstetric indication for antepartum hospitalization, and its associated risk factors, diagnosis, and management in the antepartum period are well described [1–4]. Serious morbidity associated with pyelonephritis in pregnancy is common. Sepsis and septic shock occur secondary to pyelonephritis more frequently than secondary to any other infectious process during pregnancy [5]. Acute respiratory distress syndrome complicates approximately 1–8.5% of pyelonephritis cases [6, 7]. Frequently, an admission to an intensive care unit is necessary. 

While the implications of pyelonephritis in the antepartum period are well described, there is little data about outcomes and complications when pyelonephritis occurs at the time of delivery [1–3, 8–11]. As these studies describe outcomes of pregnancies complicated by pyelonephritis, none of these describe specifically outcomes of patients who deliver during the admission during which they were diagnosed with pyelonephritis. The objective of this study was to expand on these studies, using a large nationwide data set, to estimate the medical, infectious, and obstetric complications associated with pyelonephritis at delivery.

## 2. Materials and Methods 

### 2.1. Study Design

The Nationwide Inpatient Sample (NIS) from the Healthcare Cost and Utilization Project of the Agency for Healthcare Research and Quality (AHRQ) for years 2008–2010 was queried for all delivery discharges [12]. The NIS is the largest all-payer inpatient database in the United States and contains data from approximately 8 million hospital admissions from over 1,000 hospitals in 42 states (2008) to 45 states (2010). The hospitals in the NIS are stratified based on ownership, bed size, teaching status, urban/rural location, and region. From each stratum, the NIS contains approximately 20% of United States (US) hospitals, and the sampling frame comprises 90% of all US hospital discharges. The information included in the NIS is similar to that in a typical discharge summary with safeguards to protect the privacy of individual patients, physicians, and hospitals. Weighting estimates are included for each hospitalization that allows for national estimates to be made [12]. 

Using the NIS for each of the years 2008–2010, all records containing a delivery discharge were identified. A delivery admission was defined as a hospitalization that included a delivery code (International Classification of Diseases, 9th Revision, Clinical Modification [ICD-9-CM] codes 74 for cesarean section; 72, 73, and 75 for vaginal delivery; V27 or 650–659 for nonspecific delivery codes) [13, 14]. Deliveries were also identified by diagnosis-related group (DRG) codes. DRG codes 765 and 766 were utilized to identify cesarean deliveries and codes 767, 768, 774, and 775 for vaginal deliveries. The ICD-9-CM codes used to identify discharges with pyelonephritis were 590.0–590.9. To identify comorbidities, both the ICD-9-CM code for a particular condition in pregnancy and the general ICD-9-CM codes were used (Supplemental Table 1 of the Supplementary Material available online at http://dx.doi.org/10.1155/2013/124102 for list of ICD-9-CM codes used). 

### 2.2. Statistical Analysis

All admissions in the NIS for delivery were identified. Logistic regression analyses were used to compute odds ratios with 95% confidence intervals for age, race/ethnicity, medical conditions and events, and pregnancy-related complications among women with pyelonephritis compared to women without pyelonephritis. A multivariable logistic regression model was created controlling for age, insurance status, income quartile of residing ZIP code, and the studied preexisting medical conditions to identify the odds of various medical and obstetric complications among women with pyelonephritis compared to women without pyelonephritis. Race/ethnicity was not included in the final model due to the large number of entries in the NIS with missing race/ethnicity data. Statistical significance for all analyses was assigned as a *P* value <0.05. Analyses were performed using SAS version 9.3 (SAS Institute Inc., Cary, NC) and GraphPad Prism 6.0 for Macintosh (GraphPad Software, San Diego, CA). This study protocol was reviewed and approved by the Duke University Medical Center Institutional Review Board as exempt research.

## 3. Results

During the period from 2008 through 2010, there were estimated 12,628,746 deliveries in the NIS, of those, 26,397 patient records included a diagnosis of pyelonephritis at a delivery discharge. The overall rate of pyelonephritis during delivery admission for the years 2008 through 2010 was 2.1 per 1000 deliveries. 

Table 1 describes demographic information in patients with and without pyelonephritis. Women with pyelonephritis during the delivery admission were more likely to be African American or Hispanic, were younger, less likely to have private insurance, and were more likely to reside in a ZIP code whose median income was in the lowest quartile (Table 1). Race/ethnicity data was missing for 13.8% of women with pyelonephritis and for 15.5% of women without pyelonephritis. 

Table 2 presents preexisting medical conditions associated with pyelonephritis. Women with pyelonephritis during the delivery admission were more likely to have cardiomyopathy, asthma, diabetes, systemic lupus erythematosus, anemia, thrombocytopenia, sickle cell disease/thalassemia, or chronic renal failure compared to women without pyelonephritis. Women with pyelonephritis during the delivery admission were also more likely to use drugs, alcohol, or tobacco compared to women without pyelonephritis. 

Medical complications were common among women with pyelonephritis at delivery admission. Women with pyelonephritis had increased odds of acute heart failure, deep vein thrombosis, pulmonary edema, and acute renal failure compared to women without pyelonephritis. Infection complications were also common among women with pyelonephritis. Women with pyelonephritis at a delivery admission had increased odds of pneumonia, acute respiratory distress syndrome, and sepsis compared to women without pyelonephritis. In addition, women with pyelonephritis were more likely to have a transfusion and require mechanical ventilation compared to women without pyelonephritis (Table 3).

Women with pyelonephritis were more likely to be carrying a multiple gestation or have their pregnancy complicated by preterm labor or chorioamnionitis compared to women without pyelonephritis. Cesarean delivery, gestational diabetes, preeclampsia/eclampsia/gestational hypertension, premature rupture of the membranes, and postpartum hemorrhage were all less common among women with pyelonephritis compared to women without pyelonephritis at the time of delivery admission (Table 4).

Table 5 outlines the adjusted analysis for various medical and obstetric complications at delivery while controlling for age, insurance status, income of residing ZIP code, and pre-existing medical conditions in women who had pyelonephritis at the time of delivery. Most significantly, sepsis was 108 times more likely to occur among women with pyelonephritis compared to women without pyelonephritis. Other infectious complications remained significantly associated with pyelonephritis, including pneumonia, acute renal failure, pulmonary edema, and acute respiratory distress syndrome in our adjusted model. Multiple gestation, chorioamnionitis, and preterm labor were also more common in the pyelonephritis population. 

## 4. Discussion

Pyelonephritis is the most common nonobstetric indication for antepartum hospitalization and is a common source of maternal and neonatal morbidity [3]. Here, we demonstrate that pyelonephritis at delivery admission is also common. We reconfirm several well-known preexisting medical conditions that have been associated with pyelonephritis during pregnancy and further demonstrate that women with pyelonephritis at delivery admission are at increased risk for severe infectious complications, such as pneumonia, ARDS, and sepsis, and are also at increased risk for transfusion, requiring mechanical ventilation, acute heart failure, pulmonary edema, and acute renal failure. As our study utilized data from a large database representing the entire United States, the conclusions and findings from our analysis largely validate findings from previously noted smaller, single-center studies.

It is well known that women who develop pyelonephritis during pregnancy are at increased risk of sepsis and ARDS and require ICU admission compared to nonpregnant women who develop pyelonephritis [1–3, 5, 15]. Using the NIS, it became evident that these women are at markedly increased risk for these and other complications. Women with pyelonephritis during the delivery are over 100 times more likely to have sepsis than women who do not have pyelonephritis. Pneumonia was 18 times more likely in women with pyelonephritis, and ARDS and pulmonary edema were both 11 times more likely to occur in this population. There was no difference in the risk for pulmonary emboli or deep vein thrombosis, suggesting an inflammatory mechanism for lung injury not a coagulation-related mechanism. These findings illustrate that women with pyelonephritis at delivery are at risk for significant morbidity. 

Belkin et al. utilized the NIS from a single year, 2006, to identify demographic data among women admitted at any time during pregnancy with pyelonephritis [15]. Similar to our study, they found that women less than 20 and women of African American or Hispanic race/ethnicity were at higher risk for pyelonephritis in pregnancy, though they did not compare these demographics to women without pyelonephritis. They found that women with pyelonephritis had associated risk for sepsis (2.0%), anemia (22.4%), diabetes (3.7%), and preterm labor (3.8%), but again, they did not determine the rate of these outcomes among women without pyelonephritis [15]. In our study, by analyzing data only at admissions for delivery, rather than at antepartum admission, we were able to estimate outcomes for individuals, rather than hospitalizations, as women could possibly have more than one antepartum hospitalization during a pregnancy but only a single hospitalization per woman per pregnancy. Additionally, by examining discharge data, obstetric outcomes data could be captured, which would not be available using antepartum discharges. Lastly, we were able to calculate ORs for associated complications among women with pyelonephritis since we also determined the rates of these events in pregnant women at delivery without pyelonephritis.

Our study has limitations. Our findings rely on a large discharge database, and therefore we are unable to determine if the associated preexisting medical conditions caused pyelonephritis, or if pyelonephritis was causative of the observed medical and obstetric events that were more common among women with pyelonephritis. Regardless, our data demonstrate that pyelonephritis is at least associated with these events. Next, there is no way to ascertain if coded preexisting medical conditions were active at the time of delivery. Furthermore, preexisting conditions or medical/obstetric events occurring at delivery would have been missed if the variable was not coded. Therefore, we may be underrepresenting some conditions or events. Finally, neonatal discharge records are not linked to maternal discharge records within the NIS; thus, we are unable to provide information on neonatal outcomes among those neonates born to women with pyelonephritis.

Discharge databases are also open to questions regarding the accuracy of coding. Yasmeen et al. recently assessed the accuracy and reliability of obstetric discharge databases and found them to provide helpful information [16]. Thus, despite the limitations of the NIS database, the study design allows the examination or relatively rare disorders using a large numbers of pregnancies. In doing so, odds ratios and maternal morbidity rates for medical conditions, pregnancy-related conditions, and pregnancy outcomes that would have otherwise been difficult to quantify can be estimated. Our multivariable logistic regression model attempted to identify an independent association of pyelonephritis with various medical and obstetric outcomes. In doing so, we controlled for clinically relevant preexisting medical conditions and social factors that are known to be commonly associated with pyelonephritis. In using a database such as the NIS, it is impossible to account for all potential confounders, and as such we acknowledge this limitation. 

In summary, this dataset reaffirms many of the known risk factors for pyelonephritis, including minority race, young age, lower socioeconomic status, nongestational diabetes, and sickle cell disease, but also uniquely examines preexisting medical conditions and medical and obstetric complications among women with pyelonephritis occurring at delivery. Many women with pyelonephritis at delivery were found to have other associated infectious complications such as sepsis, pneumonia, and acute respiratory distress syndrome. It is unknown if pyelonephritis was directly causative of these outcomes, and future research is warranted. 

## Supplementary Material

Supplemental Table 1: ICD-9 codes for pre-existing medical conditions, medical events, and obstetric complications utilized to identify cases in the NIS 2008 – 2010.

## Figures and Tables

**Table 1:** Demographic data at the time of delivery in hospital discharges among women with pyelonephritis compared to women without pyelonephritis, Nationwide Inpatient Sample years 2008–2010 (12,628,746 deliveries).

	Pyelo *n* = 26,397	No Pyelo *n* = 12,602,349	OR (95% CI)	*P* value
Race/ethnicity, *n* (%)				
Caucasian	9436 (35.7)	5,561,876 (44.1)	1.0	—
African American	3948 (14.9)	1,507,268 (12.0)	1.5 (1.5, 1.6)	<0.0001
Hispanic	7644 (29.0)	2,418,562 (19.2)	1.9 (1.8, 1.9)	<0.0001
Other	1738 (6.6)	1,161,608 (9.2)	0.9 (0.8, 0.9)	<0.0001
Missing	3634 (13.8)	1,953,036 (15.5)	—	—
Age, yrs^a,e^	24.9 ± 14.5	27.6 ± 13.7	—	<0.0001
Private insurance, *n* (%)^e^	7421 (28.1)	6,252,591 (49.6)	0.4 (0.4, 0.4)	<0.0001
Median house income in ZIP code of lowest quartile, *n* (%)^d,e^	9192 (34.8)	3,331,368 (26.4)	1.5 (1.5, 1.5)	<0.0001
LOS, days^b,e^	3 (2, 4)	2 (2, 3)	—	<0.0001^c^
Total charges, $^b,e^	11,175 (7030, 19,174)	10,000 (6785, 15,090)	—	<0.0001^c^

^a^Values are mean ± SD.

^b^Values are median (quartile).

^c^Wilcoxon rank sum.

^d^Median house income in ZIP code of lowest quartile defined as median income in subject's ZIP code $1–$38,999.

^e^The numbers of missing variables for each demographic descriptor are listed as follows for women with and without pyelonephritis, respectively: age (10 [0.04%] and 13,490 [0.1%]), insurance status (50 [0.2%] and 17,975 [0.1%]), house income status (475 [1.8%] and 253,740 [2.0%]), LOS (0 [0%] and 430 [0.003%]), and total charges (630 [2.4%] and 288,585 [2.3%]).

**Table 2:** Preexisting medical conditions present at time of delivery in hospital discharges among women with pyelonephritis compared to women without pyelonephritis, Nationwide Inpatient Sample years 2008–2010 (12,628,746 deliveries).

Condition, *n* (%)	Pyelo *n* = 26,397	No Pyelo *n* = 12,602,349	OR (95% CI)	*P* value
Heart disease				
Cardiomyopathy	39 (0.1)	5705 (0.04)	3.3 (2.4, 4.4)	<0.0001
Valvular heart disease	108 (0.4)	55,209 (0.4)	0.9 (0.8, 1.1)	0.513
Pulmonary disease				
Asthma	1423 (5.4)	410,193 (3.3)	1.7 (1.6, 1.8)	<0.0001
Endocrine disorders				
Diabetes (nongestational)	560 (2.1)	133,796 (1.1)	2.0 (1.9, 2.2)	<0.0001
Thyroid disorder	510 (1.9)	299,418 (2.4)	0.8 (0.7, 0.9)	<0.0001
Autoimmune disorders				
Systemic lupus erythem.	77 (0.3)	16,596 (0.1)	2.2 (1.7, 2.7)	<0.0001
Hematologic disorders				
Thrombophilia/APS	141 (0.5)	71,313 (0.6)	0.9 (0.8, 1.1)	0.516
Anemia	5406 (20.5)	1,358,360 (10.8)	2.1 (2.1, 2.2)	<0.0001
Thrombocytopenia	443 (1.7)	114,254 (0.9)	1.9 (1.7, 2.0)	<0.0001
Sickle cell/thalassemia	86 (0.3)	19,559 (0.1)	2.1 (1.7, 2.6)	<0.0001
Drugs/alcohol/tobacco				
Drug use	852 (3.2)	164,788 (1.3)	2.5 (2.3, 2.7)	<0.0001
Alcohol use	56 (0.2)	14,034 (0.1)	1.9 (1.5, 2.5)	<0.0001
Tobacco	2531 (9.6)	792,389 (6.3)	1.6 (1.5, 1.6)	<0.0001
Chronic hypertension/renal failure				
Chronic hypertension	488 (1.8)	248,440 (2.0)	0.9 (0.8, 1.02)	0.148
Chronic renal failure	40 (0.1)	5012 (0.04)	3.8 (2.8, 5.1)	<0.0001

APS: antiphospholipid antibody syndrome.

**Table 3:** Medical events present at time of delivery in hospital discharges among women with pyelonephritis compared to women without pyelonephritis, Nationwide Inpatient Sample years 2008–2010 (12,628,746 deliveries).

Condition, *n* (%)	Pyelo *n* = 26,397	No Pyelo *n* = 12,602,349	OR (95% CI)	*P* value
Transfusion	793 (3.0)	130,911 (1.0)	2.9 (2.7, 3.2)	<0.0001
Mechanical ventilation	221 (0.8)	8807 (0.07)	12.1 (10.6, 13.8)	<0.0001
Cardiac events				
Acute heart failure	67 (0.2)	4523 (0.04)	7.1 (5.5, 8.9)	<0.0001
Pulmonary events				
Pneumonia	635 (2.4)	12,589 (0.1)	24.7 (22.7, 26.7)	<0.0001
Pulmonary edema	55 (0.2)	2008 (0.02)	13.0 (9.8, 16.9)	<0.0001
Acute respiratory distress syn	88 (0.3)	2798 (0.02)	15.0 (12.1, 18.5)	<0.0001
Thromboembolic Events				
Pulmonary embolism	11 (0.04)	3609 (0.03)	1.3 (0.6, 2.3)	0.405
Deep vein thrombosis	22 (0.08)	6352 (0.05)	1.6 (1.03, 2.4)	0.022
Infections				
Sepsis	1393 (5.3)	5426 (0.04)	129 (122, 137)	<0.0001
Renal event				
Acute renal failure	257 (1.0)	6828 (0.05)	18.1 (15.9, 20.5)	<0.0001

**Table 4:** Obstetric events present at time of delivery in hospital discharges among women with pyelonephritis compared to women without pyelonephritis, Nationwide Inpatient Sample years 2008–2010 (12,628,746 deliveries).

Condition, *n* (%)	Pyelo *n* = 26,397	No Pyelo *n* = 12,602,349	OR (95% CI)	*P* value
Obstetric events				
Cesarean delivery	3669 (13.9)	4,037,688 (32.0)	0.3 (0.3, 0.3)	<0.0001
Operative vaginal delivery	645 (2.4)	791,598 (6.3)	0.4 (0.3, 0.4)	<0.0001
Multiple gestation	1392 (5.3)	265,839 (2.1)	2.6 (2.4, 2.7)	<0.0001
GDM	982 (3.7)	714,105 (5.7)	0.6 (0.6, 0.7)	<0.0001
Prex, eclamp, gest HTN	1271 (4.8)	929,959 (7.4)	0.6 (0.6, 0.7)	<0.0001
Preterm labor	3696 (14.0)	1,048,007 (8.3)	1.8 (1.7, 1.9)	<0.0001
Premature rupture of membranes	440 (1.7)	469,115 (3.7)	0.4 (0.4, 0.5)	<0.0001
Postpartum hemorrhage	375 (1.4)	321,599 (2.5)	0.5 (0.5, 0.6)	<0.0001
Chorioamnionitis	1285 (4.9)	322,282 (2.5)	1.9 (1.8, 2.1)	<0.0001

Eclamp: eclampsia; GDM: gestational diabetes; gest HTN: gestational hypertension; prex: preeclampsia.

**Table 5:** Multivariable logistic regression analysis for the listed outcomes among women with pyelonephritis compared to women without pyelonephritis, while controlling for age, private insurance, residing in a ZIP code with annual income at lowest quartile, cardiomyopathy, asthma, diabetes, thyroid disorders, systemic lupus erythematous, anemia, thrombocytopenia, sickle cell disease, drug use, alcohol use, tobacco use, chronic hypertension, and chronic renal failure.

	Adjusted OR (95% CI)	*P* value
Transfusion	1.8 (1.7, 2.0)	<0.0001
Mechanical ventilation	10.9 (9.5, 12.5)	<0.0001
Cardiac events		
Acute heart failure	6.5 (4.9, 8.5)	<0.0001
Pulmonary events		
Pneumonia	18.5 (17.0, 20.1)	<0.0001
Pulmonary edema	11.3 (8.6, 14.9)	<0.0001
Acute respiratory distress syndrome	11.6 (9.3, 14.3)	<0.0001
Thromboembolic events		
Pulmonary embolism	1.1 (0.6, 2.0)	0.858
Deep vein thrombosis	1.6 (1.02, 2.4)	0.038
Infections		
Sepsis	108 (101, 115)	<0.0001
Renal event		
Acute renal failure	14.7 (12.9, 16.7)	<0.0001
Obstetric events		
Cesarean delivery	0.3 (0.2, 0.4)	<0.0001
Operative vaginal delivery	0.4 (0.3, 0.4)	<0.0001
Multiple gestation	2.9 (2.8, 3.1)	<0.0001
GDM	0.7 (0.7, 0.8)	<0.0001
Prex, eclamp, gest HTN	0.6 (0.5, 0.6)	<0.0001
Preterm labor	1.7 (1.5, 1.8)	<0.0001
Premature rupture of membranes	0.4 (0.4, 0.5)	<0.0001
Postpartum hemorrhage	0.4 (0.4, 0.5)	<0.0001
Chorioamnionitis	1.7 (1.6, 1.8)	<0.0001

Eclamp: eclampsia; GDM: gestational diabetes; gest HTN: gestational hypertension; Prex: preeclampsia.
