# Supplementary material for: Medical and Infectious Complications Associated with Pyelonephritis among Pregnant Women at Delivery
Source: Infect Dis Obstet Gynecol. 2013 Sep 28;2013:124102. doi: 10.1155/2013/124102 (PMC3804393; doi:10.1155/2013/124102)
Supplement: Supplementary file 1 — Supplemental Table 1: ICD-9 codes for pre-existing medical conditions, medical events, and obstetric complications utilized to identify cases in the NIS 2008 – 2010. [file 124102.f1.pdf]

Supplemental Table 1. ICD-9 codes for pre-existing medical conditions, medical events, and obstetric complications utilized to identify cases in the NIS 2008 – 2010.

**Medical Condition**	**ICD-9 codes**
**Heart Disease**	
Cardiomyopathy	425.x, 674.5
Valvular heart disease	394-397, 424, 785.2, 785.3, V42.2
**Pulmonary Disease**	
Asthma	493
**Endocrine**	
Diabetes (non-gestational)	249, 250, 648.0
Thyroid disease	240-246, 648.1
**Autoimmune**	
Systemic lupus erythema.	710.0, 695.4, 583.8
**Hematologic**	
Thrombophilia (includes history of thrombosis and antiphospholipid syndrome [APS])	273.8, 286.53, 286.9, 289.81, 289.82, 289.9, 795.79, V12.51
Anemia	648.2x, 285.x
Thrombocytopenia	287.3x, 287.4x, 287.5x
Sickle cell/thalassemia	282.4-282.49, 282.6-282.69
**Drug/Alcohol/Tobacco**	
Drug use	292, 304, 305.2x-305.9x, 655.5x, 760.70, 760.72-760.75, 779.5, 965.0x, V65.42

Alcohol use	291, 303, 305.0x, 760.71, 980.0x
Smoking	305.1, V15.82, 649.0x
**Chronic hypertension/renal failure**	
Chronic Hypertension	401-405, 437.2, 642.0-642.2
Chronic Renal Failure	585, 792.5, V42.0, V45.1, V56
**Event or Condition**	
Mechanical Ventilation	Procedure codes: 93.90, 96.01-96.05, 96.7x
Transfusion	Procedure codes: 99.00-99.09
**Cardiac Event**	
Acute heart failure	428
**Pulmonary Event**	
Pneumonia	480-486, 487.0
Pulmonary edema	518.4
ARDS	518.5
**Thromboembolic Event**	
Pulmonary embolism	415.1x, 673.x
Deep venous thrombosis	451.1x, 451.2x, 671.3x, 671.4x
**Infections**	
Sepsis	038, 790.7
**Renal Event**	

Acute renal failure	584, 639.3x, 669.3x
**Obstetric Events**	
Cesarean delivery	Procedure codes: 74, DRG 765, DRG 766
Operative vaginal delivery	Procedure codes: 720-724, 726, 727-727.9
Multiple Gestation	651, 652.6
Gestational diabetes	648.8x
Preeclampsia, eclampsia or gestational hypertension	642.3-642.7
Preterm labor	644
Premature rupture of membranes	658.1x
Postpartum hemorrhage	666-666.2x
Chorioamnionitis	658.4x, 659.2x
